# Assessment of Electrosensitivity of the Pulp of the Mandibular Second Molar after Surgical Removal of an Impacted Mandibular Third Molar

**DOI:** 10.3390/jcm10163614

**Published:** 2021-08-16

**Authors:** Grzegorz Trybek, Magda Aniko-Włodarczyk, Olga Preuss, Aleksandra Jaroń

**Affiliations:** Department of Oral Surgery, Pomeranian Medical University in Szczecin, 72 Powstańców Wlkp. St., 70-111 Szczecin, Poland; dominika.wlodarczyk@pum.edu.pl (M.A.-W.); olga.preuss@pum.edu.pl (O.P.); jaronola@gmail.com (A.J.)

**Keywords:** impacted third molar, pulp sensitivity, mandibular third molar, complications, impaction, surgical removal

## Abstract

Despite the frequent discussion of complications associated with surgical removal of wisdom teeth in the scientific literature, increased mobility of the second molar, which can affect the clinical status of the pulp, is often downplayed or overlooked. This study aimed to evaluate surgical removal of an impacted third molar on the change in the electrosensitivity of the pulp of the mandibular second molar. Sixty patients consecutively presenting to the Department of Oral Surgery to remove an impacted mandibular third molar were included in the study. Clinical examinations of pulp sensitivity of second molars in both the study and control groups were evaluated before the procedure, seven days after the procedure, and eight weeks after the procedure. The surgical removal of an impacted mandibular third molar significantly affected the pulp sensitivity of the second molar.

## 1. Introduction

The impacted mandibular third molar is characterized by considerable variability in the morphological structure and time of the eruption in the alveolar arch. The surgical extraction of the wisdom tooth is the most frequent procedure in dental surgery [1,2]. Like any other surgery, it involves the risk of complications including tissue swelling, trismus, pain, infection, lip and tongue sensory deficits as well as damage to an adjacent tooth [3]. The age, gender of the patient, the degree of retention of the impacted tooth, and the operator’s experience affect the appearance of complications associated with the surgical removal of an impacted mandibular third molar [4,5,6]. Complications prolong the postoperative recovery period and cause swelling, trismus, or inflammatory complications and may eventually lead to clinical changes in the mandibular second molar region [7].

Despite the frequent discussion of complications associated with surgical removal of wisdom teeth in the scientific literature, increased mobility of the second molar, which can affect the clinical status of the pulp, is often downplayed or overlooked [8].

To assess the pulp status of the tooth before and after surgery, a tooth vitality test can be used to provide valuable diagnostic information and influence the treatment plan. Assessment of pulp sensitivity can be performed using an EPT—electric pulp test [9]. Eugene Russell Ziegler developed the first pulp vitality test device in 1953. He patented it in the USA seven years later, where it was assigned patent number 2949107 [10]. The dental pulp is a highly innervated tissue, mainly by Aβ, Aγ, and Aδ core fibers and non-core C, which is grouped according to diameter and conduction rate. Core A fibers originate from Gasser’s ganglion, whereas C fibers are part of the vegetative system. Ninety percent of the nerve fibers in the dental pulp are Aδ fibers, which, along with blood vessels, enter the root canal and tooth chamber through the apical opening [11,12,13]. Electrical impulses stimulate the peripherally located myelinated Aδ fibers of the pulp–dentin complex [14]. The electrical stimulation causes ionic shifts across the neuron membrane, inducing an action potential with rapid spikes of depolarization between the Ranvier constrictions of the myelinated fiber [15]. Nonmyelinated C fibers, located centrally, have a higher sensitivity and are not stimulated. EPT indicates efficiently conducting pulp nerve fibers [16,17]. A valuable pulp sensitivity testing device is the PEm-1-type pulpoendometer (Narol-Dental, Warsaw, Poland). The apparatus consists of a body and two electrodes: a passive one, attached to the patient’s lip, and an active one, applied to the examined tooth. The location of the active electrode on the tooth under examination has a significant influence on the value and repeatability of the measurement. To increase the effectiveness of the measurement, the probe should be located in the area with the highest concentration of Aδ nerve fibers and minimum thickness of enamel and dentin. In young subjects, this is the pulp corner, and in older subjects, it is the region of the tooth neck [18,19]. In the case of molars, one-third of the buccal surface height of the tooth is recommended. The device displays measurement values on a scale from 0 to 99, expressed in degrees. The test is based on the pain response of the pulp to a stimulus, which is a faradic current. Its intensity for a healthy pulp does not exceed 40 µA [20]. The response to stimulation with electrical impulses of the lowest voltage determines the pulp sensitivity (ps). The ps value should be compared with an opposing tooth to interpret the results objectively. For molar teeth, a range of reference values from 40° to 80° is considered the norm for pulp excitability. The test cannot be performed on teeth which have a surface that is entirely covered by a filling or prosthetic crown. Trauma to the second mandibular molar affects the pulp sensitivity value. Aδ fibers are susceptible to oxygen deficit and stop functioning correctly before pulp necrosis occurs. Clinically, this manifests itself by the tooth not responding to the EPT despite its vitality, i.e., a false negative result. Examination of the pulp with an electrical test is more effective in assessing viability than diagnosing necrosis [21]. Correctly performed, it is a safe clinical test that provides valuable information about the dental pulp. It is reliable in assessing the dental pulp condition immediately after surgical trauma and allows for monitoring its condition over time [22,23].

The complications of surgical extraction of impacted wisdom teeth in the form of impaired blood supply to the pulp of the second molar, which can lead to pulp necrosis, are overlooked in the literature. There is a particular lack of data on the effect of the surgical procedure on changes in the threshold of excitability of the pulp of the second molar and, thus, its clinical condition.

This study aimed to evaluate surgical removal of an impacted third molar on the change in the electrosensitivity of the pulp of the mandibular second molar.

## 2. Materials and Methods

After obtaining approval from the Bioethics Committee number KB-0012/89/16 of Medical University, the study was conducted in the Department of Oral Surgery.

The inclusion criteria were age over 18 years, presence of second and third molars on the right and left sides in the mandible, no general diseases, no permanent medication, and non-smokers who signed informed consent to participate in the study. The exclusion criteria were age below 18 years, general conditions, malocclusion, subjects undergoing orthodontic treatment, mandibular second molars undergoing endodontic treatment and with large fillings, smokers, and patients with implanted cardiac pacemakers. 

**Figure 1:**
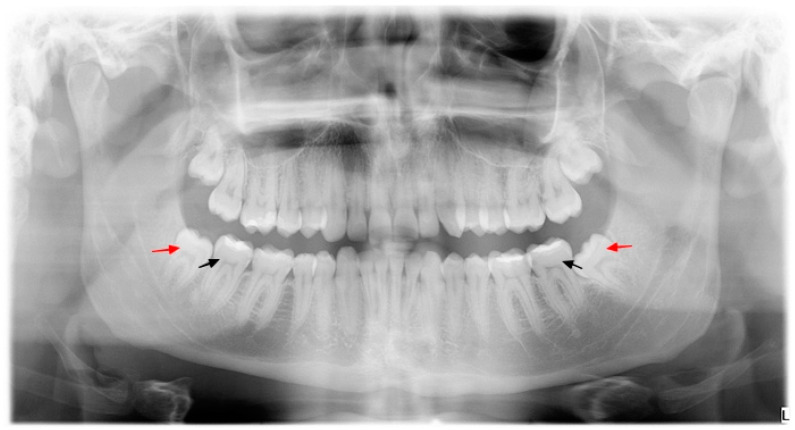
Orthopantomogram of a patient in the study group; red arrow—mandibular third molar, black arrow—mandibular second molar.

A total of 120 teeth were examined: a right and a left mandibular second molar in each patient (Figure 1). All available patients meeting the inclusion and exclusion criteria, who applied to the clinic from May 2017 to June 2017, by convenience sampling, were considered to be participants in the study. The examined teeth were divided into two groups—60 teeth in each group:

Study group (*n* = 60)—second mandibular molars on the operated side;

Control group (*n* = 60)—mandibular second molars on the opposite side of the mandible.

Oral surgery specialists with a similarly high level of surgical experience performed all procedures. Tooth extraction was performed under local anesthesia (2% Lignocainum with noradrenaline) in the amount of 4–6 mL. The procedure started with exposing the impacted third mandibular molar by making a full-thickness flap. The tooth was removed using a drill and Bein’s straight elevator and/or Meissner’s universal/root forceps. The wound was closed with single knotted sutures, which were left in place for seven days. Patients were advised to rinse their mouths with a 0.1% chlorhexidine solution and to take nonsteroidal anti-inflammatory drugs (100 g ketoprofen, twice daily).

### 2.1. Clinical Examination

Pulp sensitivity was analyzed using a Narol-Dental PEm-1 pulpoendometer. Before the examination, the patient was instructed to signal the sensation of the stimulus by raising the left hand. The buccal surface of the tooth was isolated from the moist oral environment with gauze swabs. An active electrode, moistened with sterile water, was applied to a point determined by the intersection of a vertical line running in the middle of the buccal surface width of the clinical crown and a horizontal line running in one-third of the buccal crown height. After obtaining confirmation from the patient about the sensation of the stimulus, the result was read on the device display. The recorded result was compared with a scale representing the average pulp sensitivity values for healthy molars. An analogous test was performed for the tooth on the opposite side of the arch. Each lower second molar was tested twice, and the mean value of both measurements was used for statistical analysis.

### 2.2. Methodology of Statistical Analysis

Qualitative variables were described by the number and percentage of occurrences of each value. Arithmetic means, standard deviation, median, quartiles, and minimum and maximum values of qualitative variables were calculated.

The chi-square test was used to compare the qualitative variables in the study and control groups. To make a more accurate comparison between groups, the multiple comparisons method for post hoc analysis (Fisher’s exact test for small expected values) was used. For binary variables, the analysis of qualitative variables’ values in two repeated measurements were performed using the McNemar test. Both in the case of the presence or absence of normal distribution, Bonferroni correction was applied. The significance level adopted in the study was 0.05. If the *p*-value was below that level, it was interpreted as statistically significant. 

## 3. Results

### 3.1. Baseline Characteristics

Sixty patients—17 males and 44 females—participated in the study. The mean age of the study subjects was 24.82 years (±5.51). Table 1 summarizes the detailed results of the study sample. The spatial location of impacted third molars in the mandible was determined using Winter and Pell and Gregory’s classifications [6]. The position of the impacted teeth is shown in Table 2. 

### 3.2. Comparative Analysis of the Response to Electric Current of Second Molars of the Study Group and the Control Group 

#### 3.2.1. Pre-Treatment Measurement

In the study group, all teeth responded appropriately to the current test. In the control group, three teeth did not respond to pulp testing. The differences between the study and control groups were not significant (*p* > 0.05). The rest of the data were collected and are presented in Table 3.

#### 3.2.2. Measurement Seven Days after Treatment

Seven days after treatment, a significant increase in the number of teeth in the study group showed no response (from 0 to 19) to the electrical test (*p* = 0.001) compared to the control group. This represented 31.67% of the teeth in the study group. There were four cases of no response in the control group. The remaining statistical data are shown in Table 4.

#### 3.2.3. Measurement Eight Weeks after the Treatment

Eight weeks after treatment, there were no statistically significant differences in response to the electrical stimulus of the teeth of the test and control groups (*p* > 0.05). There were four instances of no response to electrical stimulation in the test group and three in the control group. The results are shown in Table 5.

### 3.3. Assessment of the Threshold of Excitability of the Second Molar Measured with a Pulpometer before the Procedure, Seven Days after the Procedure and Eight Weeks after the Procedure

Sequential tests showed a significant increase in the percentage of unresponsive teeth to EPT after seven days compared to before treatment and a significant decrease in unresponsive teeth eight weeks after treatment (*p* < 0.001). The results of the analysis are shown in Table 6.

### 3.4. Comparative Analysis of the Change in the Pulp Sensitivity Measurements of Second Molars in the Study and Control Groups between Individual Time Points Measured with a Pulpometer

At time interval 0 → 1, a significant increase in the number of teeth unresponsive to EPT was observed in the study group compared to the control group. In the time interval 1 → 2, the emergence of response was more frequent in the study group than in the control group. The values of the analysis performed are shown in Table 7.

### 3.5. Measurement of Second Molar Electrosensitivity Seven Days after Surgery and Eight Weeks after Surgery, as Measured by Pulpometer concerning the Predicted Difficulty of Surgery

The number of mandibular second molars that showed no response to faradic current was not statistically significantly related to the difficulty of the mandibular wisdom tooth removal procedure determined by Pederson’s classification at any time point (*p* > 0.05). The highest number of teeth (*n* = 14) not responding to the EPT test was after seven days among patients after a moderately difficult procedure. The results are summarized in Table 8.

## 4. Discussion

The electrical examination of the dental pulp provides valuable diagnostic information about its condition. Combined with information gathered from the history and clinical analysis of the tooth, it is the basis for treatment planning [15]. Pulp quality assessment based on qualitative sensory response is widely used in monitoring its condition after trauma.

Surgical removal of an impacted wisdom tooth in the mandible traumatizes the adjacent second molar, affecting the sensitivity of its pulp. In our study, pulp sensitivity, measured by an electrical test, of second molars seven days after surgery showed its lack of response to stimulus in as many as 31.67% of the teeth of the study group. This represents a significant increase in the number of teeth with pulp unresponsive to EPT after surgical removal of lower wisdom teeth in the study group compared to the control group (*p* = 0.001). In contrast, eight weeks after the procedure, the teeth responded adequately to the electrical test, and the difference between the groups was not statistically significant (*p* > 0.05).

One-third of the teeth seven days after the procedure did not respond to the electrical test. Underlying this phenomenon is the pulp shock the second lower molar is in after surgical removal of the wisdom tooth in the mandible [24]. The trauma affects the periapical tissues and the area of the cervical border where the pulp chamber of the tooth is located [25]. The lack of response to the EPT test may be a natural response of the pulp of the second lower molar to surgical intervention.

However, it is noteworthy that before the surgical procedure, all of the study teeth responded adequately to faradic current. In contrast, eight weeks after surgery, the pulp of the four second mandibular molars in the study group still did not respond to the electrical test. This is clinically significant because such teeth require monitoring after trauma. The surgical removal of an impacted lower wisdom tooth leads to partial damage to the supporting tissues of the second molar, which is clinically analogous to subluxation of the tooth. Usually, the teeth do not require endodontic treatment but only observation and waiting for the return of normal response to stimuli [26]. However, in the few cases where the normal pulp response does not return, according to Andreasen et al., there is a fifty percent risk of pulp necrosis within six months [27]. There is a wealth of information in the literature about monitoring the pulp status of teeth after trauma and about possible therapeutic management. These can be applied to the pulp of second molars after surgical removal of wisdom teeth.

Examination of the pulp’s response to an electrical stimulus, combined with a clinical exam confirming a color change, a positive percussion test, allows the diagnosis of pulp necrosis. To the authors’ knowledge, there are no studies in the literature monitoring the pulp response of second molars to an electrical viability test before and after surgical removal of wisdom teeth in the mandible.

In 2016, Oguz et al. published the need for root canal treatment of second molars as a complication arising after surgical removal of an impacted third mandibular molar and maxilla [28]. The authors evaluated the medical records of 6232 patients presenting for surgical removal of a wisdom tooth. Only eleven cases required root canal treatment. Of interest is that the incidence of complications was estimated at 0.17% in the mandible only. In our study material, unresponsiveness was characterized by 6.67% of teeth after eight weeks. Due to the short observation period, the clinical status of the four teeth showing no response to faradic current examination is yet to be clarified. If pulp necrosis occurred, the incidence rate ratio of this type of complication would be significantly higher in the conducted study than that presented by Oguz et al. [28]. The difference is that this was a retrospective study, and the authors analyzed the available medical records. It is uncertain whether patients with an indication for root canal treatment of a second molar did not take treatment at another clinic. Patients already presented to the surgical clinic with symptoms from the periapical tissues. Since the pulp sensitivity was not monitored before and after the surgical procedure, the pulp status was also unknown [28].

The influence of procedures from other fields, such as orthognathic or traumatology, from maxillofacial surgery, as well as the influence of forces released during orthodontic treatment on the pulp electrosensitivity of teeth near the area subjected to the procedures mentioned above, has been described [29,30,31].

Brajdić et al. conducted a viability study of 459 teeth in 50 patients after osteosynthesis of mandibular fracture. A three-year follow-up period showed a lack of response to pulp faradic current in 8% of the teeth tested. In three cases, a decision was made for root canal treatment due to the appearance of clinical signs of pulp necrosis [29]. In mandibular fractures, teeth’s lack of pulp response to EPT testing is more complex and multifactorial. The lack of response may be due to the different fracture lines (there may be several), extent and degree of injury, inferior alveolar nerve damage, and other surgical scenarios.

Vedtofte and Nattestad conducted a study of the viability of 617 teeth in 51 patients before and after a Le Fort I osteotomy procedure [32]. The procedure consisted of jaw displacement in either the horizontal or vertical plane, depending on the previous surgical scenario. Pulp necrosis was diagnosed in three teeth within the maxilla, representing 0.5% of all teeth examined. Taub et al. estimated the incidence of pulp necrosis after osteotomy procedures in the mandible to be 7% to as high as 50%, depending on the surgical technique used [33].

Romanos et al. described single cases of dental pulp necrosis after open sinus floor elevation surgery [30]. In the three cases described by the authors, pulp necrosis occurred after three months, after six months, and after thirty months, respectively. In the last case, it was debatable whether the surgery was the cause of necrosis because of the long time interval.

It is noteworthy that for 36% of patients, orthodontic indications are the reason for presenting for surgical removal of an impacted wisdom tooth [34]. As reported in a study by Guanghong et al., tooth movement caused by orthodontic forces can cause severe pulp changes associated with blood flow disorders. Teeth during orthodontic treatment may temporarily respond negatively to electrical tests [34]. This is another reason why it would be advantageous to introduce into the preoperative diagnosis of lower molar removal in the mandible—electrosensitivity testing of teeth in the vicinity of the treatment field. This will avoid diagnostic and treatment errors concerning the second lower molar.

Temporary lack of response to stimuli is a common symptom during post-traumatic pulp healing, especially after post-traumatic tooth dislocation. Because of the results obtained, we conclude that one of the irreversible complications of surgical removal of the impacted lower wisdom tooth may be loss of pulp viability of the second molar. The electrical pulp vitality test is the best tool in long-term monitoring [35].

According to Cunha-Crus et al., 79% of patients presenting for surgical removal of a wisdom tooth are referred by dentists to prevent future complications related to the presence of the impacted tooth rather than because of current pathology [36].

Luyten et al. evaluated the pulp sensitivity of palatally impacted maxillary canines treated with an open or closed surgical exposure technique. Overall, as many as more than 20% of canines were nonresponsive after exposure surgery [37].

The present study results indicate the need for evaluation of the clinical status of the second molar before surgery and periodic monitoring after removal of the impacted third mandibular molar. The assessment should examine parameters such as probing depth, mobility, gingival index, and pulp excitability threshold. This management algorithm allows for minimizing complications associated with the second molar, the clinical status of which is often overlooked in diagnosing and treating complications after surgical removal of an impacted wisdom tooth in the mandible.

A limitation of the study is the small study group. Future studies are planned on a more extensive study group. Moreover, in the study, the pulp sensitivity of the second mandibular molar could be determined by different methods. In addition, researchers could prolong the follow up. 

The pulp sensitivity study of the second molar could influence the clinical decisions made by the dentist in the diagnostic and treatment process. Because of the transient decrease in pulp sensitivity, dentists should defer endodontic treatment decisions for the second molar, which, because of trauma, may erroneously indicate the need for endodontic treatment after surgical removal of an impacted mandibular third molar.

## 5. Conclusions

The surgical removal of an impacted mandibular third molar significantly affects the pulp sensitivity of the second molar. After seven days, more than 30% of the mandibular second molars showed no reaction in the EPT test. This was a transient condition; after eight weeks, only slightly more than 6% showed no response to the EPT test. The number of mandibular second molars that did not show a response to the EPT test was not statistically significantly related to the difficulty of the mandibular second molar removal.

## Figures and Tables

**Table 1:** Characteristics of the study patients.

	Age		Sex	
			Female	Male
Mean (±SD)	28.82 (±5.51)	*n*	43	17
Median	23			
Quartile 1	21	%	71.67	28.33
Quartile 3	38			

SD—standard deviation, *n*—number of subjects.

**Table 2:** Characteristics of the position of lower wisdom teeth.

Classification								
Winter			Pell and Gregory					
			Depth of Impaction			Relation to the Anterior Margin of the Mandibular Ramus		
	*n*	%		*n*	%		*n*	%
Mesial	30	50.00	level A	30	50.00	class 1	9	15.00
Horizontal	7	11.67	level B	19	31.67	class 2	40	66.67
Vertical	16	26.67	level C	11	18.33	class 3	11	18.33
Distal	7	11.67						

*n*—number of patients.

**Table 3:** Comparison of tooth excitability responses of the test group and the control group before treatment.

Response to the EPT Test before Treatment	Study Group		Control Group		*p* *
	*n*	(%)	*n*	(%)	
No reaction (−)	0	0.00	3	5.00	0.248
Reaction (+)	60	100.00	57	95.00	

* McNemar test; *n*—number of teeth, *p*—significance level.

**Table 4:** Comparison of the response of second molars of the study group and the control group to electrical test 7 days after treatment.

Response to the EPT Test after 7 Days	Study Group		Control Group		*p* *
	*n*	(%)	*n*	(%)	
No reaction (−)	19	31.67	4	6.67	0.001
Reaction (+)	41	68.33	56	93.33	

* McNemar test; *n*—number of teeth, *p*—significance level.

**Table 5:** Comparison of the response to the electrical stimulus of the teeth of the test group and the control group 8 weeks after treatment.

Response to the EPT Test after 8 Weeks	Study Group		Control Group		*p* *
	*n*	(%)	*n*	(%)	
No reaction (−)	4	6.67	3	5.00	1
Reaction (+)	56	93.33	57	95.00	

* McNemar test; *n*—number of teeth, *p*—significance level.

**Table 6:** Percentage of unresponsive teeth to EPT at each time point.

	No Reacting		Reacting		Significance of Change *	
	*n*	(%)	*n*	(%)	Versus Previous Measurement	Versus before the Surgery
Before the surgery	0	0.00	60	100.00	–	–
7 days after the surgery	19	31.67	41	68.33	<0.001	<0.001
8 weeks after the surgery	4	6.67	56	93.33	0.001	0.134

* McNemar tests with Bonferroni correction.

**Table 7:** Comparison of the change in the threshold of excitability of the pulp of second molars in the test and control groups between individual time points as measured by a pulpometer.

Change *		Study Group (*n =* 60)		Control Group (*n* = 60)		*p* **
		*n*	(%)	*n*	(%)	
0 → 1	loss of reaction	19	31.67	1	1.67	<0.001
	no change	41	68.33	59	98.33	
	appearance of reactions	0	0.00	0	0.00	
0 → 2	loss of reaction	4	6.67	0	0.00	0.119
	no change	56	93.33	60	100.00	F
	appearance of reactions	0	0.00	0	0.00	
1 → 2	loss of reaction	1	1.67	0	0.00	<0.001
	no change	43	71.67	59	98.33	F
	appearance of reactions	16	26.67	1	1.67	

* 0—before treatment; 1–7 days after treatment; 2–8 weeks after treatment; ** Chi-square test, F = Fisher’s exact test (low expected values in table); *n*—number of teeth, *p*—significance level.

**Table 8:** Comparison of the relationship between the predicted difficulty of the procedure and the electrosensitivity measurement of the mandibular second molar seven days after the procedure and eight weeks after the procedure as measured by a pulpometer.

7 Days after the Surgery	Slightly Difficult (*n* = 10)		Moderately Difficult (*n* = 36)		Very Difficult (*n* = 14)		p *
	*n*	(%)	*n*	(%)	*n*	(%)	
No reaction	2	20.00	14	38.89	3	21.43	0.424
Reaction	8	80.00	22	61.11	11	78.57	
8 weeks after the surgery							
No reaction	0	0.00	2	5.56	2	14.29	0.347
Reaction	10	100.00	34	94.44	12	85.71	

* Fisher’s exact test (low expected values in table); *n*—number of teeth, *p*—significance level.

## Data Availability

Data available on request.
